# Fluid shifts are main drivers for microgravity simulation-induced immune-physiological changes: findings from the VIVALDI studies

**DOI:** 10.1038/s41526-025-00555-z

**Published:** 2026-02-16

**Authors:** Dominique Moser, Marie-Pierre Bareille, Angelique van Ombergen, Marion Hoerl, Federico D´Amico, Matthias Feuerecker, Christopher Dächert, Sandra Matzel, Adrien Robin, Nastassia Navasiolava, Marc-Antoine Custaud, Alexander Choukér

**Affiliations:** 1 Laboratory of Translational Research ‘Stress and Immunity’, Department of Anesthesiology, LMU Hospital, Ludwig-Maximilians-University Munich, Munich, Germany; 2 Institute of Space Physiology and Medicine (MEDES), Toulouse, France; 3 European Space Agency (ESA), Noordwijk, The Netherlands; 4 Max von Pettenkofer Institute and Gene Center, Virology, National Reference Center for Retroviruses, Faculty of Medicine, LMU München, Munich, Germany; 5 Université Angers, CRC, CHU Angers, Inserm, CNRS, MITOVASC, Equipe CARME, SFR ICAT, F-49000 Angers, France; 6 Aerospace and Extreme Environment Nursing Program, College of Nursing, Texas A&M University, Bryan, TX USA; 7 Paul Sabatier University, Toulouse, France; 8 University of Colorado, Aurora, CO, USA; 9 University Jean Monnet, Saint Etienne, France; 10 Montpellier University, Montpellier, France; 11 CHU Toulouse, Toulouse, France; 12 King’s College London, London, UK; 13 University of Portsmouth, Portsmouth, UK; 14 Université de Caen Normandie, Caen, France; 15 Université de Strasbourg, Strasbourg, France; 16 Université de Reims Champagne-Ardenne, Reims, France

**Keywords:** Immunology, Physiology

## Abstract

Microgravity strongly affects human physiology during spaceflight. Biological sex has not yet been sufficiently considered as a variable for spaceflight deconditioning. The VivalDI studies investigated physiological systems affected by 5-days dry immersion (DI) in females and males, with a focus on immune changes in this report. In both sexes proportions of peripheral granulocytes and NK cells were elevated during DI and T-cell numbers were reduced. Leukocyte activation and cytokine levels were moderately affected. Females showed a higher Torque-Teno-virus shedding at the end of DI. Noradrenaline concentrations increased during the study with sex-specific patterns. Hemodynamics suggest that immunological changes were caused by DI-induced fluid shifts. Moreover, male study participants’ patterns were compared to a historical data set from a 5-days head-down-tilt bed rest (HDT-BR) study. Changes in leukocyte proportions and body fluid indicators were stronger in DI versus HDT-BR. These analyses indicate that fluid shifts primarily drive intervention-related immune-physiological differences, independent of biological sex. ClinicalTrials.gov, TRN: NCT05043974 and NCT05493176.

## Introduction

Being in space, astronauts are forced to adapt to a variety of new and mostly unexperienced conditions, summarized as the space exposome^1,2^. Among these conditions, microgravity is regarded as the one having a major impact on the human immune system, which has been verified by ex vivo and in vitro investigations. Studies both in real and simulated microgravity demonstrated altered leukocyte subset distributions^3,4^, a pro-inflammatory secretory phenotype of monocytes^4,5^, Th1-Th2 shifts^6^, and reduced activation capacities of T cells^5,7^. Consequently, induced immune dysfunctions can lead to hyper-reactive immune states, resulting in hypersensitive or autoimmunological reactions^6,8^ or to hypo-reactivities, potentially increasing the incidence of infections^4^ or viral reactivation^9,10^.

In the light of planned future deep space exploration missions, a thorough understanding of the impact of the space exposome in general, and microgravity in special, is crucial to prevent increased susceptibilities to disease and in the case of illness, to apply efficient countermeasures. Despite increasing specificity and personalization of prescribed countermeasures in recent years^11^, biological sex has remained an underexplored variable. Generally, women exhibit a more robust immune response to foreign and self-antigens compared to men^12^. They have higher CD4^+^ T cell counts and CD4/CD8 ratios as well as B cell numbers and immunoglobulin levels, resulting in higher pathogen clearance in case of infection and a higher vaccine efficiency. But these potent immune reactions have been also shown to increase the susceptibility of developing inflammatory and autoimmune diseases^3,12–14^.

To date, fewer than 12 % of space crew member have been female^15^. However, female astronauts are planned to be increasingly enrolled in future long-term deep space missions such as returning to the moon or traveling to Mars, which is already reflected by the gender-mixed composition of the Artemis II crew. Thus, elaborating on sex-specific differences regarding immunological adaptations to the space exposome is indispensable.

Research opportunities on human physiology aboard the ISS are limited, and the investigation of sexual-based differences in adaptation processes is significantly constrained by the disproportionate representation of female and male crew members.

Dry immersion (DI) is a valuable model to mimic conditions of spaceflight-associated microgravity by supportlessness, achieved by buoyancy-induced full unloading of the body^15–17^. Within DI, study subjects are immersed up to the neck in a thermoneutral water bath, whereby the body is separated from water by a waterproof fabric^15,17^. Due to hydrostatic compression of almost the whole body, body fluids are redistributed in a headward direction^18^, comparable to situations in spaceflight^19^. The comparative *Integrative Study of Physiological Changes Induced by a 5-Day Dry Immersion in Healthy Female and Male Volunteers* (VivalDI I&II) was initiated to characterize for the first time sex-specific alterations in human physiology and psychology induced by five days DI. Because of the known sexual dimorphism in immune cells distributions and functions, a differential impact of a DI intervention on the immune state in females and males was suggested. The present report summarizes the outcome of these investigations with focus on immune state and neuroendocrine stress parameters of the study participants.

Additionally, and in preparation of further studies to explore effects of whole-body microgravity simulation, data derived from the VivalDI II group (males) was contrasted with results obtained from an all-male 5-days head-down tilt bed-rest (HDT-BR) study as another well-established model to study physiological deconditioning simulated microgravity.

## Methods

### 5-days dry immersion study design

Samples and data were obtained in the scope of *Integrative Study of Physiological Changes Induced by a 5-Day Dry Immersion on Healthy Female Volunteers* (VivalDI I) and *A 5-day Dry Immersion Study on Healthy Male Volunteers* (VivalDI II) [ClinicalTrials.gov Identifiers: NCT05043974 and NCT05493176]. The participation in this study was voluntary and every study participant gave her/his informed consent. Applied procedures and techniques were performed in accordance to the Declaration of Helsinki and approved by the National Ethic Committee (CPP Ile de France II: 5 July 2021, no. ID RCB: 2021-A00705-36 for Vivaldi I; CPP Ile de France VII: 20 June 2022, no. ID RCB: 2022-A00881-42 for Vivaldi II) and French Health Authorities (ANSM: 31 May 2021 for Vivaldi I; ANSM: 24 May 2022 for Vivaldi II).

Each trial involved the recruitment of 20 participants. One female participant left the protocol on the first day of immersion due to a technical issue, and another could not be included for regulatory reasons. One male participant withdrew from the study on the third day of immersion due to severe back pain. The study was completed by 18 women and 19 men. Demographic data are summarized in Table 1.

**Table 1:** Demographic data of female (*n* = 18) and male (*n* = 19) VivalDI study participants

			*P* value
Subject number	18	19	–
Age [years]	28.8 ± 4.7	28.0 ± 4.3	0.714^a^
Weight [kg]	59.3 ± 6.3	72.0 ± 6.7	**<0.001**^**b**^
BMI [kg.m^2^]	21.8 ± 1.8	23.1 ± 1.9	0.051^b^

Values are given as mean ± SD.

Differences between groups were calculated by Mann–Whitney U test^a^ or unpaired two-tailed Student’s *t* test^b^; significant *P* values are indicated in bold.

The study was conducted at the MEDES space clinic, Toulouse, France from 20 Sept. 2021 to 10 Dec. 2021 (VivalDI I; females) and from 20 Sept. 2022 to 23 Nov. 2022 (VivalDI II; males). The study protocol consisted of 4 days ambulatory baseline data collections before DI (BDC-4 - BDC-1), five days of DI (DI1–DI5) and 2 days of ambulatory recovery (R0, R+1). The complete study protocol was published previously^15^. For the present investigations, samples were collected at BDC-1, DI3, DI5 and R+1.

### 5-days head-down tilt bed rest study for comparative analyses in males

To assess distinct impacts of DI and HDT-BR for the relevant time points before intervention (BDC), on day 3, day 5 and after intervention (return), data sets from male VivalDI II study participants were compared to historical data (*n* = 12) generated within a 5-days 6° HDT-BR study that included male study participants only. This study was conducted at MEDES in 2010/2011 (BRAG-2, ID-RCB number: 2008-A00576-49). Data from the HDT-BR study was published previously by Feuerecker et al.^20,21^. Participants of the DI and HDT-BR study groups slightly differed in age (Table 2).

**Table 2:** Demographic data of male participants in the dry immersion (DI) study (*n* = 19) and the head-down tilt bed rest (HDT-BR) cohort (*n* = 12)

	DI	HDT-BR	*P* value
Subject number	19	12	
Age [years]	28.0 ± 4.3	32.6 ± 7.1	**0.033**^**b**^
Weight [kg]	72.0 ± 6.7	75.1 ± 7.6	0.240
BMI [kg.m^2^]	23.1 ± 1.9	24.0 ± 1.9	0.171

Values are given as mean ± SD.

Differences between groups were calculated by unpaired two-tailed Student’s *t* test^b^; significant *P* values are indicated in bold.

### Blood and saliva sampling

Venous blood samples were collected in the morning via forearm venipuncture from the study participants. Whole blood was fixated with Transfix (Cytomark, Buckingham, UK) and stored at 4 °C until phenotyping, but not longer than 14 days. Aliquoted serum and plasma samples were stored at −80 °C until further analysis. Saliva samples were collected in the morning and evening before tooth brushing and food ingestion using a Salivette (Sarstedt, Nümbrecht, Germany). Additional saliva samples were collected in 13 ml collection tubes. The samples were stored at −80 °C until analysis.

### White blood cell count and blood parameters

Complete blood count was performed at the MEDES space clinic, Toulouse, France with Automated Hematology & Coagulation analyzers. Hemoglobin was measured by HemoCue Hb-201® and hematocrit by microcentrifugation. Plasma volume changes were assessed by Hb-Hct Dill & Costill formula^22^ with referring to BDC values as control. C-reactive protein concentrations in serum were measured by Immuno-turbidimetry - Advia chemistry XPT (Siemens, Munich, Germany).

### Staining of cell surface molecules and flow cytometry

For analysis of leukocyte subset proportions, BD Biosciences™ IMK-Kit was used (Cat. No. 340182, BD Biosciences, Franklin Lakes, NJ, USA). Activation states of leukocytes in whole blood were assessed by the relative surface expression of relevant activation markers by staining with fluorochrome-conjugated antibodies. For monocytes, leukocytes were stained for co-expression of CD14 and CD40, CD69, CD80, CD86, CD11b, TLR2, TLR4 and HLA-DR. T cells were stained for expression of CD69 and CD28 on CD4^+^ T helper cells and CD8^+^ cytotoxic T cells. Activation state of CD16^+^ granulocytes was examined by analysis of CD62L, CD11b and CD66b expression. Except for TLR2 (130-120-052, Miltenyi Biotec, Bergisch Gladbach, Germany) and TLR4 (FAB6248C, R&D Systems, Minneapolis, MN, USA), all antibodies were purchased from BD Biosciences (Cat. No. CD4:555346, CD8:555369, CD11b: 550019, CD16:338440, CD14: 345784, CD28: 337181, CD40: 555589, CD62L: 555544, CD66b: 555724, CD69: 555531, CD80: 557227, CD86: 555665, HLA-DR: 559866, Franklin Lakes, NJ, USA). For immune cell staining 25 µl of blood samples were mixed with antibodies and incubated for 20 min in the dark, followed by 10 min lysis (BD FACS lysing solution, BD Biosciences, Franklin Lakes, NJ, USA) and two subsequent washing steps with PBS. Per samples 10,000 events were measured by flow cytometry (Guava® easyCyte™ 8HT Flow Cytometer, Merck Millipore, Billerica, MA, USA) and data analysis was performed with InCyte Software for Guava® easyCyte™ HT Systems (Merck Millipore).

### Cytokine measurements

Concentrations of EDTA plasma cytokines IL-1β, IL-1Ra, IL-8, IL-10, TNF, IFNγ, G-CSF, MIP-1α, and MIP-1β were quantified using the MAGPIX Multiplexing System (Luminex, Austin, TX, USA) with custom-made multiplex assays (Merck Millipore, Darmstadt, Germany) according to the manufacturer’s instructions.

### ELISAs

Quantification of soluble CD62L in plasma was performed using the sL-Selectin ELISA kit (Cat. No. 30150496, Tecan IBL International, Hamburg Germany). ELISA Kits for both dehydroepiandrosterone (DHEA; Cat. No. SLV-3012) and dehydroepiandrosterone sulfate (DHEA-S; Cat. No. SLV-4409) in saliva were obtained from DRG Instruments (Marburg, Germany). All assays were carried out according to the manufacturers’ instructions. The optical densities for all assays were measured by Emax Plus plate reader (Molecular Devices, San Jose, CA, USA) at 450 nm and analyzed by SoftMax Pro 7 software (Molecular Devices, San Jose, CA, USA).

### EBV and TTV viral load determination

Viral loads of the stress marker viruses Epstein-Barr-Virus (EBV) and Torque-Teno-virus (TTV) were determined for BDC-1 and R+1 timepoints in the accredited routine diagnostics laboratory of the Max von Pettenkofer Institute, virology department according to established standard operating procedures. First, nucleic acids from saliva were extracted on a QIAsymphony SP instrument using the QIAsymphony DSP Virus/Pathogen Mini Kit (Qiagen, Hilden, Germany). In-house qPCRs were then used to assess EBV and TTV viral loads. PCR reactions were performed with LightCycler® 480 Probes Master mix (Roche Diagnostics, Mannheim, Germany) on a LightCycler® 480 Instrument II. Plasmid dilutions served to generate standard curves for absolute quantification. Factor 2 regarding TTV being single-stranded DNA vs. standard plasmid was neglected.

### Stress hormones measurements – salivary cortisol

Cortisol concentrations were determined in a blinded manner by electrochemiluminescence immunoassay (Elecsys 2010; Roche, Mannheim, Germany) at the Institute of Clinical Chemistry, LMU Hospital, Munich, Germany. Concentrations of dehydroepiandrosterone and dehydroepiandrosterone sulfate were determined by ELISA (see section ELISAs).

### Stress hormones measurements - urine catecholamines and cortisol

Urinary adrenaline and noradrenaline concentrations were measured from 10 ml samples derived from 24-h urine collections. Quantification of concentrations was performed by high-performance liquid chromatography (Chromosytems, Martinsried, Germany) at the Institute of Clinical Chemistry, LMU Hospital, Munich, Germany. The absolute mass of catecholamines in urine samples was calculated from the overall urine catecholamine concentration and the urine volume of the respective collection period. Free urinary cortisol was measured through liquid chromatography-tandem mass spectrometry (Applied Biosystems/MDS Sciex Api 3000, Waltham, MA, USA).

### Statistics

Statistical data analysis was performed with the commercially available software SPSS statistics 29 (IBM, Armonk, NY, USA) and graphs were created with SigmaPlot 13 (Systat, Erkrath, Germany). Statistical significance of demographic data was tested by unpaired two-tailed Student’s *t* test or by Mann–Whitney *U* test when data was not normally distributed. Group- and time-dependent mixed effects were tested by mixed ANOVA followed by Bonferroni correction. Interaction and main effect terms as well as group differences, pre- and post-differences and Cohen’s d of significantly different pairwise comparisons over time are summarized in the Supplementary Tables 1 and 2. *P* < 0.05 was regarded as statistically significant. Single missing data was estimated by linear interpolation which is indicated together with sample size in the figure legends.

## Results

### Comparison of DI-related effects in women and men – proportions of leukocyte subsets

**Fig. 1:**
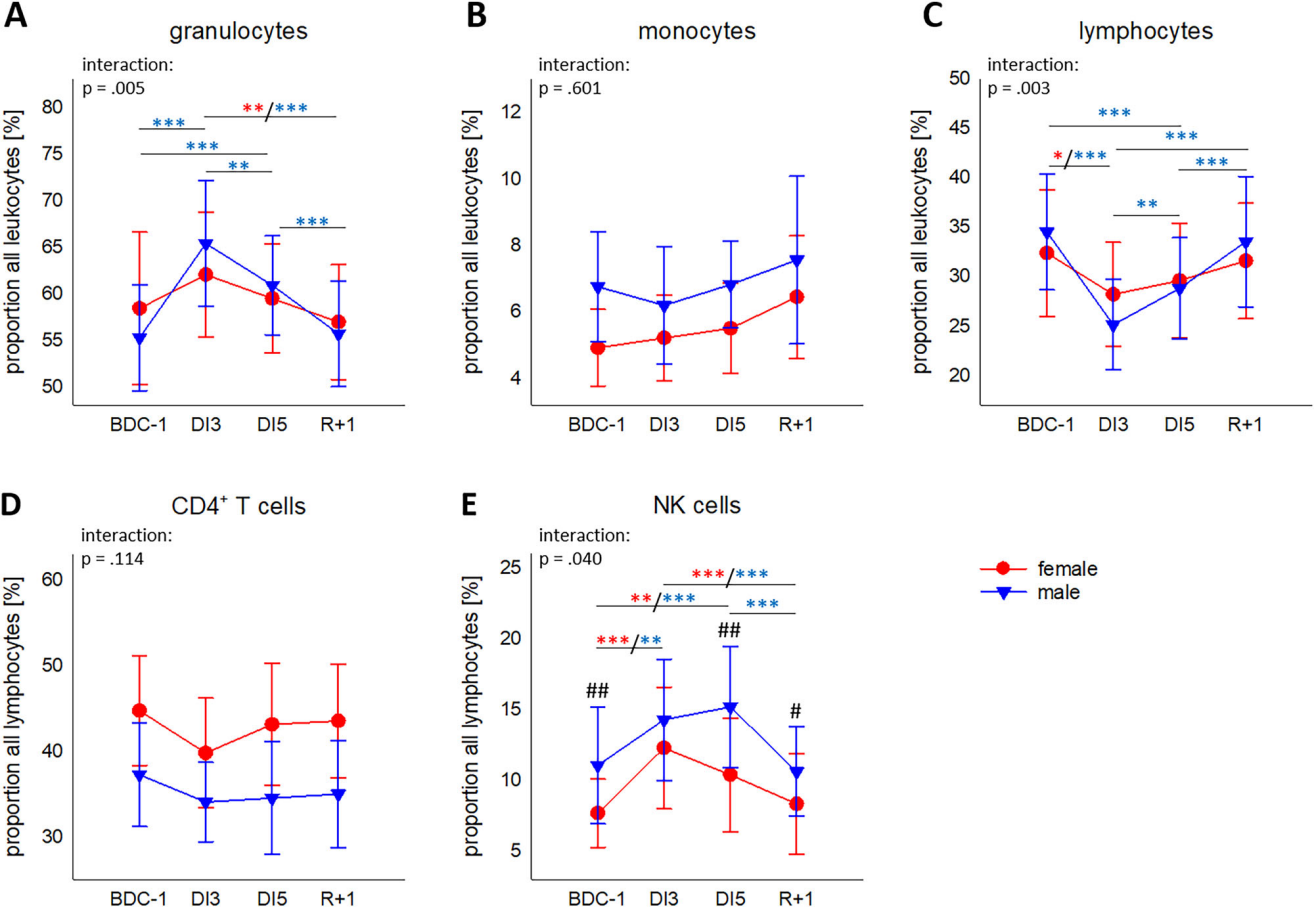
Changes in leukocyte proportions during DI. Proportions of granulocytes (**A**), monocytes (**B**) and lymphocytes (**C**) among all leukocytes in whole blood as well as of CD4^+^ T cells (**D**) and NK cells (**E**) among all lymphocytes. Plots represent mean values ± SD in females (red, *n* = 18) and males (blue, *n* = 19) over the courses of observation times. Data was analyzed with mixed ANOVA followed by Bonferroni post hoc test in the case of significant interaction. Differences between groups: ^#^*P* < 0.05, ^##^*P* < 0.01. Differences between time points within the same group: *P < 0.05, **P < 0.01, ***P < 0.001, whereby differences in the female group are indicated in red and in the male group in blue asterisks. Single missing values were estimated by linear interpolation.

Granulocyte proportions showed a significant interaction and were increased in both sexes during DI with a peak at day 3 (Fig. 1A, Supplementary Table 1). Monocyte proportions were higher in males than in females but remained at a constant level during DI (Fig. 1B). Lymphocyte proportions likewise displayed significant interaction but, opposite as granulocytes, proportions declined during DI with a nadir at DI3 (Fig. 1C, Supplementary Table 1). Proportions of CD4^+^ T cells appeared higher in females than in males, but both groups showed no alterations in time course (Fig. 1D). Proportions of CD8^+^ T cells showed no differences between groups or time points (not shown). NK cells displayed significant interaction with a pattern comparable to granulocytes and proportions rose in both sexes during DI. However, while a peak was reached in females already at DI3, highest values for males were measured at DI5. Both increases were followed by a decline to BDC-1 levels at R+1. Males had higher NK cells proportions among lymphocytes than females (Fig. 1E, Supplementary Table 1).

Measured proportions comply with absolute values of white blood cell count by automated Hematology & Coagulation analyzers (Table 3, Supplementary Table 1).

**Table 3:** Absolute numbers of granulocytes (neutrophils), monocytes and lymphocytes per µl blood

	Neutrophils/µl		Monocytes/µl		Lymphocytes/µl	
					
BDC-1	2975.2 ± 822.7	2912.1 ± 733.4	476.7 ± 123.0	529.5 ± 116.1	2215.4 ± 477.0	1981.1 ± 256.0
DI3	**4022.1** ± 1058.2^***^	**4383.7** ± 1069.5^***^	499.4 ± 137.0	580.0 ± 151.4	2053.1 ± 433.1	1771.1 ± 292.4
DI5	**4190.4** ± 1519.6^***^	**3643.2** ± 894.4^*^	517.5 ± 152.1	597.4 ± 126.4	2122.7 ± 524.8	1933.2 ± 293.2
R+1	**3527.7** ± 855.4^**^	2996.3 ± 609.9^#^	568.5 ± 137.2	640.0 ± 135.3	2210.5 ± 400.4	1954.7 ± 296.1

Values are given as mean ± SD. Females: *n* = 18; males: *n* = 19.

Data was analyzed with mixed ANOVA followed by Bonferroni post hoc test in the case of significant interaction (*p* = 0.006 for neutrophils). Differences between groups: ^#^*P* < 0.05. Differences between time points within the same group: *P < 0.05, **P < 0.01, ***P < 0.001. Significant difference to BDC is indicated in bold.

### Activation states of leukocytes

In addition to analyses of the leukocyte proportions, their activation states were examined by measuring expression levels of cell-type specific activation markers. On CD14^+^ monocytes, expression levels of CD40 declined continuously during DI in females and remained at a low level at R+1. In males, expression levels were slightly increased at DI3 and DI5. Comparable trends were also observed for CD69, CD80 and TLR4. For CD86, remarkable increases were detected in females and CD11b was higher at DI5 in females and at all timepoints in males. An interaction effect was not observable for these surface markers on monocytes. Expression of TLR2 and HLA-DR remained stable over the study course (Table 4, upper rows).

**Table 4:** Relative cell surface expression of cell activation markers on CD14^+^ monocytes, CD4^+^ and CD8^+^ T cells as well as on CD16^+^ granulocytes

		DI3		DI5		R+1	
					
CD14	CD40	0.77 ± 0.39	1.15 ± 0.53	0.69 ± 0.35	1.20 ± 0.52	0.69 ± 0.47	0.92 ± 0.49
	CD69	0.69 ± 0.58	1.10 ± 0.42	0.78 ± 0.50	1.08 ± 0.40	0.82 ± 0.73	0.83 ± 0.41
	CD80	0.73 ± 0.57	1.06 ± 0.43	0.73 ± 0.39	1.13 ± 0.54	0.72 ± 0.64	0.90 ± 0.39
	CD86	1.10 ± 0.21	1.04 ± 0.20	1.23 ± 0.34	1.07 ± 0.35	1.18 ± 0.23	1.11 ± 0.28
	CD11b	1.04 ± 0.27	1.32 ± 0.70	1.50 ± 1.01	1.28 ± 0.75	0.96 ± 0.27	1.29 ± 0.54
	TLR2	1.03 ± 0.05	1.00 ± 0.02	1.03 ± 0.04	1.00 ± 0.02	1.02 ± 0.05	1.00 ± 0.02
	TLR4	0.87 ± 0.48	1.12 ± 0.33	0.79 ± 0.40	1.23 ± 0.58	0.60 ± 0.33	0.86 ± 0.46
	HLA-DR	1.05 ± 0.10	1.01 ± 0.04	1.04 ± 0.10	1.02 ± 0.05	1.05 ± 0.12	1.04 ± 0.07
CD4	CD69	1.21 ± 0.66	1.30 ± 0.89	0.95 ± 0.56	1.51 ± 0.76	1.10 ± 0.66	1.51 ± 1.23
	CD28	0.95 ± 0.06	1.01 ± 0.13	0.93 ± 0.08^#^	1.00 ± 0.13	**0.91** ± 0.07^**,##^	1.02 ± 0.13
CD8	CD69	1.24 ± 0.78	1.42 ± 1.27	0.91 ± 0.51^**#**^	**1.79** ± 1.20^**^	0.94 ± 0.47	1.48 ± 1.32
	CD28	**0.91** ± 0.09^**,#^	0.99 ± 0.09	**0.92** ± 0.08^**^	0.96 ± 0.11	**0.95** ± 0.08^*,#^	1.00 ± 0.08
CD16	CD62L	0.69 ± 0.69	1.41 ± 1.31	1.49 ± 1.41	0.94 ± 0.97	1.66 ± 1.69	1.34 ± 1.05
	CD11b	1.02 ± 0.89	1.38 ± 1.39	1.69 ± 1.81	0.94 ± 0.71	0.91 ± 0.60	1.46 ± 1.67
	CD66b	1.02 ± 0.08	1.00 ± 0.01	1.00 ± 0.06	0.99 ± 0.02	0.99 ± 0.10	1.00 ± 0.01

Data was normalized to the respective BDC values and shown as mean ± SD. Females: *n* = 9–18; males: *n* = 18.

Data was analyzed with mixed ANOVA followed by Bonferroni post hoc test in the case of significant interaction (*p* = 0.016 for CD4/CD28; *p* = 0.032 for CD8/CD28; *p* = 0.034 for CD8/CD28). Differences between groups: ^#^*P* < 0.05, ^##^*P* < 0.01. Differences between time points within the same group: *P < 0.05, **P < 0.01. Significant difference to BDC is indicated in bold.

Both on CD4^+^ and CD8^+^ T cells, expression of CD69 was slightly enhanced in females at DI3 compared to BDC-1, but declined again at DI5 and R+1. In males, expression was remarkably increased at DI3, DI5 and R+1, reaching significant interaction for CD8^+^ T cells with a significant increase at DI5. Expression of CD28 showed significant interaction on both T cell subsets with a decrease in females at DI3, DI5 and R+1 compared to BDC, while expression was unaffected in males (Table 4, middle rows).

Expression of surface markers was not significantly altered in granulocytes (CD16^+^). But females showed an increase in CD62L expression at DI5 and R+1 after an initial decline at DI3. In males, levels slightly increased at DI3 but declined in the further course. Similar patterns were observed for CD11b. Expression of CD66b remained unchanged (Table 4, lower rows).

### Soluble pro- and anti-inflammatory mediators

**Fig. 2:**
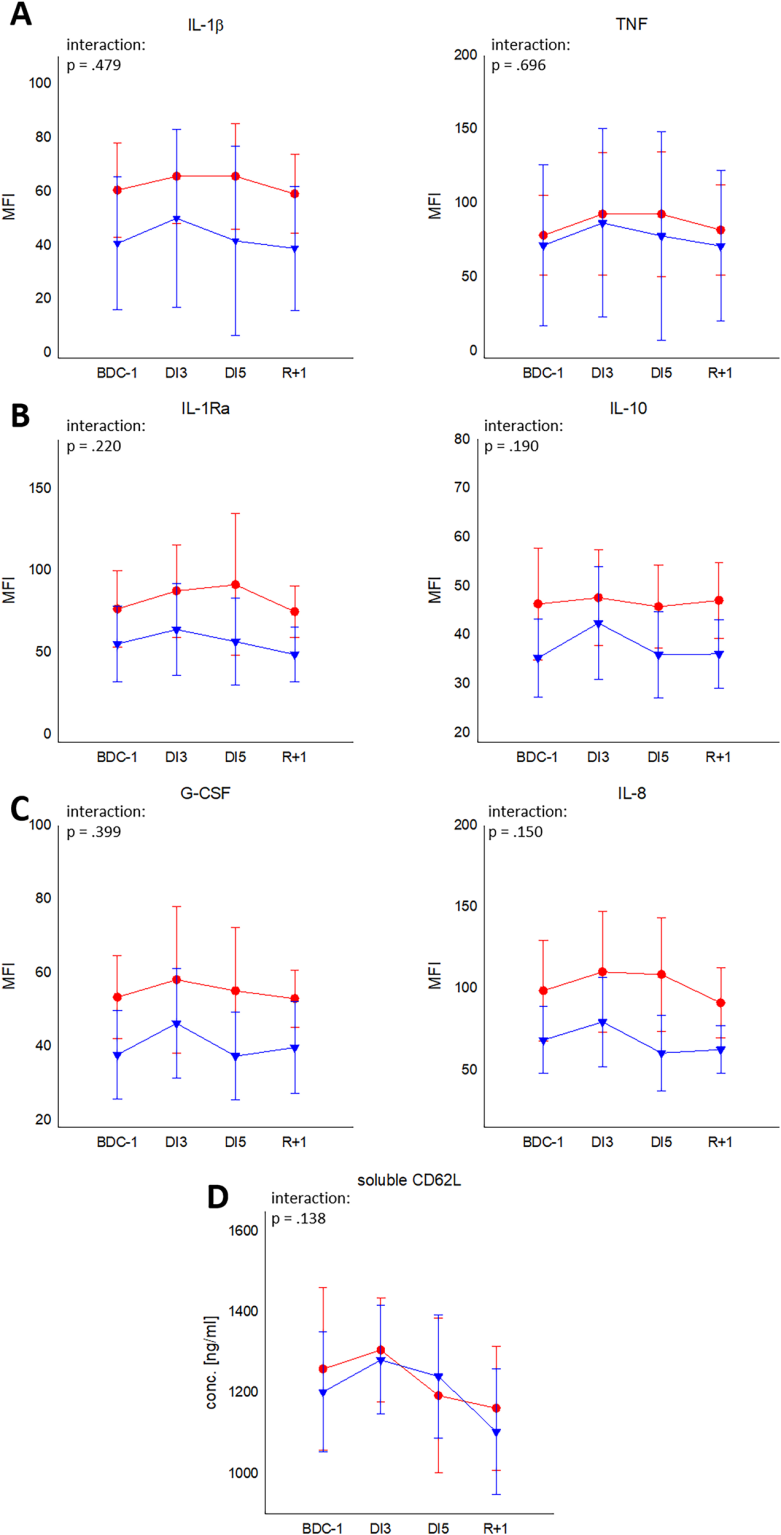
Cytokine abundance in plasma. Plasma abundance (mean fluorescence intensity (MFI)) of pro-inflammatory cytokines IL-1β and TNF (**A**), the compensatory anti-inflammatory cytokines IL-1Ra and IL-10 (**B**), the granulocyte recruiting/activating cytokines G-CSF and IL-8 (**C**) as well as concentration [ng/ml] of the inflammation marker sCD62L (**D**). Plots represent mean values ± SD in females (red, *n* = 18) and males (blue, *n* = 19) over the courses of observation times.

To further assess the immune state during DI, an extensive panel of pro- and anti-inflammatory cytokines was measured. Levels of the pro-inflammatory cytokines IL-1β, TNF, MIP-1α and MIP-1β, which are predominantly assigned to monocytes and macrophages, were slightly increased during DI (Fig. 2A, Table 5). The anti-inflammatory cytokines IL-1Ra and IL-10 also displayed increased levels at DI3, which appeared more pronounced in males for IL-10 (Fig. 2B). The granulocyte- activating cytokine G-CSF as well as the granulocyte attractant IL-8 showed both for females and males slightly increased levels at DI3 (Fig. 2C). Levels of IL-1β and its antagonist IL-Ra, as well as IL-10, G-CSF, and IL-8 were higher in females than in males, but without reaching statistical significance (Fig. 2A–C). Abundance of soluble L-selectin (sCD62L) was likewise increased at DI3 with a drop at R+1 in both sexes (Fig. 2D). The level of the T cell cytokine IFNγ remained unchanged (Table 5). For all measured cytokines, no interaction effects were observed between groups and time points (Supplementary Table 1). CRP values were at low levels throughout the study for both groups (0.2–0.4 mg/l, Supplementary Fig. 1).

**Table 5:** MFI (mean fluorescence intensity) of cytokine abundance of MIP-1α, MIP-1β, and IFNγ

	BDC		DI3		DI5		R+1	
							
MIP-1α	43.78 ± 9.60	43.79 ± 27.60	44.17 ± 10.23	55.16 ± 38.96	45.61 ± 12.24	49.05 ± 37.80	42.83 ± 9.31	44.00 ± 28.21
MIP-1β	91.17 ± 23.63	84.26 ± 29.12	106.89 ± 27.53	104.68 ± 35.45	108.17 ± 29.13	97.21 ± 38.94	99.72 ± 28.08	89.79 ± 32.76
IFNγ	327.78 ± 185.67	234.26 ± 172.263	380.44 ± 194.00	282.26 ± 209.83	394.39 ± 203.09	211.68 ± 218.32	296.39 ± 164.80	195.84 ± 150.17

Values are given as mean ± SD. Females: *n* = 18; males: *n* = 19.

Overall, exposure of female and male individuals to a 5-day DI induced an initial slight increase of pro-inflammatory immune parameters, which was however located at low levels. This effect appears to be more pronounced in males than in females, but both sexes recovered quickly within the course of DI.

### Virus shedding

Intensity of viral load of the stress marker viruses EBV and TTV was examined before start of DI and at R+1. Within the analyzed study participants (12 females and 15 males), 2 out of 12 females (16.6 %) showed an increased viral shedding of EBV at R+1 compared to BDC and 3 out of 15 males (20 %). Interestingly, 8 out of 12 females (66.6 %) displayed an increased shedding of TTV at R+1, while this occurred in 3 out of 15 males (20 %) only.

### Stress hormones

Morning levels of the steroid hormones cortisol, DHEA and DHEA-S showed no differences between females and males and remained stable at physiological ranges during the whole observation period (Table 6). Urinary cortisol quantifications after 24 h collection confirmed stability of concentration levels (Supplementary Fig. 2).

**Table 6:** Morning salivary concentrations of the steroid hormones cortisol, DHEA and DHEA-S

	BDC-1		DI3		DI5		R+1	
							
Cortisol (µg/dl)	0.58 ± 0.24	0.64 ± 0.26	0.60 ± 0.27	0.66 ± 0.25	0.60 ± 0.34	0.54 ± 0.28	0.63 ± 0.17	0.59 ± 0.17
DHEA (pg/ml)	413.53 ± 196.83	436.83 ± 213.56	393.17 ± 166.29	430.16 ± 177.33	362.40 ± 169.74	394.32 ± 252.38	358.12 ± 197.77	374.32 ± 210.23
DHEA-S (ng/ml)	5.97 ± 3.52	7.36 ± 4.21	6.16 ± 3.84	7.09 ± 3.68	6.02 ± 3.69	7.82 ± 4.44	7.48 ± 4.71	8.48 ± 4.47

Values are given as mean ± SD. Females: *n* = 17; males: *n* = 19.

**Fig. 3:**
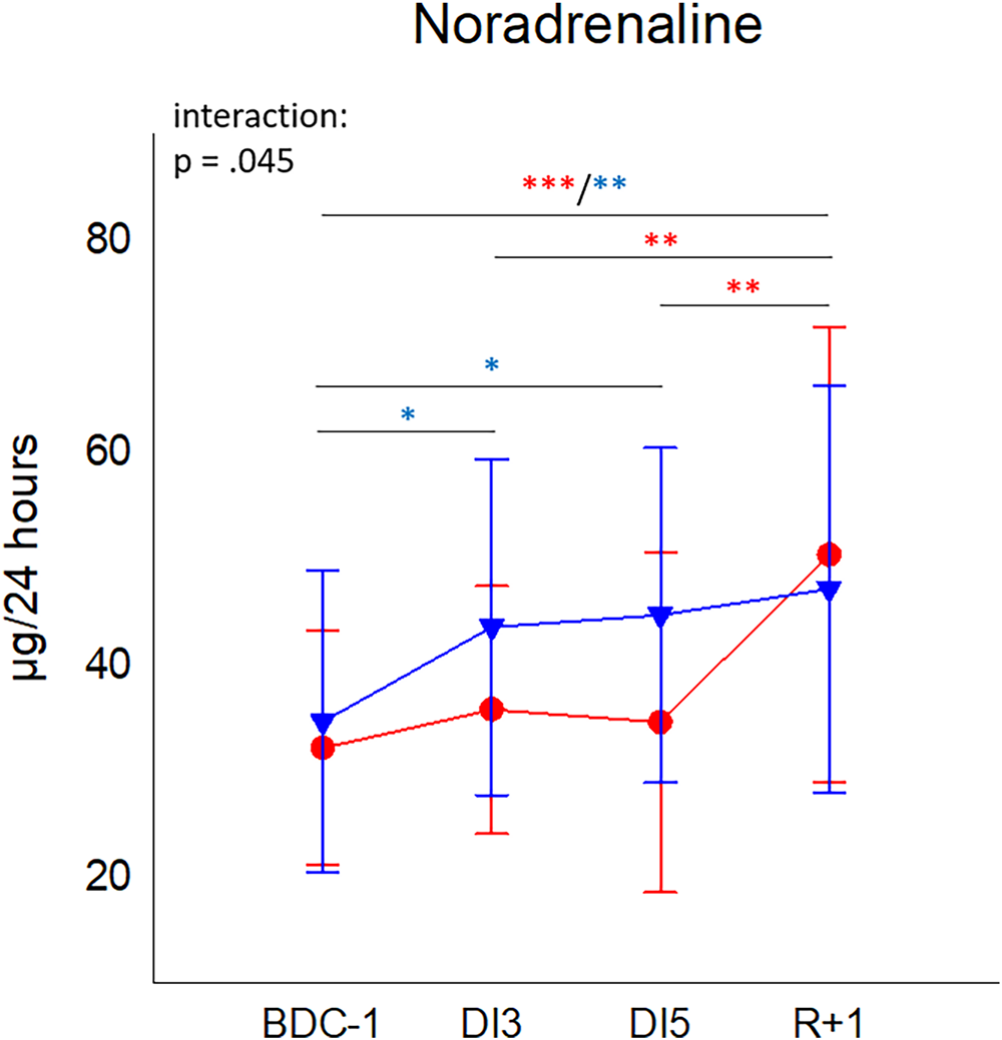
Urinary noradrenaline concentrations. Plot represent mean values ± SD in females (red, *n* = 18) and males (blue, *n* = 19) over the courses of observation times. Data was analyzed with mixed ANOVA followed by Bonferroni post hoc test. Differences between time points within the same group: *P < 0.05, **P < 0.01, ***P < 0.001, whereby differences in the female group are indicated in red and in the male group in blue asterisks. Single missing values were estimated by linear interpolation.

Concentration levels of noradrenaline were at comparable levels and displayed significant interaction with an increase in both sexes. However, while concentrations rose immediately at DI3 and stayed significantly elevated in males over the observation period, they remained low in females during DI but then increased significantly at R+1 (Fig. 3, Supplementary Table 1). Release of adrenaline remained stable during the whole study both in females and in males, whereby males displayed higher values than female study participants (Supplementary Fig. 3).

### Fluid shifts and hypovolemia

For both groups, hemoglobin, hematocrit and MCV values stayed within their respective sex-specific physiological ranges. Hemoglobin and hematocrit showed a significant interaction in time course. Compared to BDC-1, values were significantly increased in both groups at DI3 and DI5 and dropped below BDC-1 levels at R+1 (Table 7, Supplementary Table 1). Plasma volume (PV) changes amount to −20.5 ± 5.1 % (DI3) and −17.6 ± 7.0 % (DI5) in females and −24.8 ± 5.9 % (DI3) and −23.3 ± 7.4 % (DI5) in males.

**Table 7:** Blood parameters hemoglobin, hematocrit and mean corpuscular volume (MCV) as well as water intake, urine output and water balance

	BDC-1		DI3		DI5		R+1	
							
Hemoglobin (g/dl)	13.27 ± 0.65	15.55 ± 0.87	**15.16** ± 0.74^***^	**18.05** ± 0.99^***^	**14.87** ± 0.87^***^	**17.86** ± 1.00^***^	**12.79** ± 0.93^*^	15.17± 0.85
Hematocrit (%)	39.53 ± 1.91	44.85 ± 2.12	**45.19** ± 2.31^***^	**51.98** ± 2.62^***^	**44.34** ± 2.73^***^	**51.58** ± 2.72^**^	**38.33** ± 2.63^*^	**43.65** ± 2.30^*^
MCV (fl)	92.05 ± 3.72	90.58 ± 3.13	92.22 ± 3.57	90.79 ± 3.08	92.05 ± 3.42	90.68 ± 3.15	91.93 ± 3.34	90.26 ± 3.18
Water Intake (ml/24 h)	3602.28 ± 744.57	3563.90 ± 634.90	**2741.72** ± 530.09^***^	3271.90 ± 470.32^##^	**2870.67** ± 316.12^***^	**2700.58** ± 387.22^***^	3679.11 ± 688.08	3557.68 ± 651.87
Urine Output (ml/24 h)	2747.22 ± 719.62	2499.26 ± 684.01	**2285.44** ± 427.59^**^	2811.53 ± 518.36^##^	2427.89 ± 418.96	2265.21 ± 449.90	2604.00 ± 689.02	2210.00 ± 766.07
Water Balance (ml/24 h)	855.06 ± 216.38	1064.63 ± 365.23^#^	**456.28** ± 214.15^***^	**465.63** ± 192.03^***^	**442.78** ± 171.46^***^	**435.53** ± 213.38^***^	1075.11 ± 356.83	**1347.74** ± 304.59^*,#^

Values are given as mean ± SD. Females: *n* = 18; males: *n* = 19.

Data was analyzed with mixed ANOVA followed by Bonferroni post hoc test in the case of significant interaction (*p* = 0.003 for hemoglobin; *p* = 0.004 for hematocrit; *p* < 0.001 for water intake; *p* < 0.001 for urine output, *p* = 0.036 for water balance). Differences between groups: ^#^*P* < 0.05, ^##^*P* < 0.01. Differences between time points within the same group: *P < 0.05, **P < 0.01, ***P < 0.001. Significant difference to BDC is indicated in bold. Differences between groups for hemoglobin and hematocrit were significant (*p* < 0.001) for all time points (not depicted in table).

Both groups had a lower water intake during DI compared to BDC-1, reaching significant differences for DI3 in females and for DI5 in females and males. Urine output remained at comparable levels throughout the study, resulting in a relatively higher output than intake at DI3 and DI5, consequently causing lower water balance values (=intake − output values) (Table 7, Supplementary Table 1).

### Comparison of DI- and HDT-BR-related effects in men – Leukocyte subsets and granulocyte activation

**Fig. 4:**
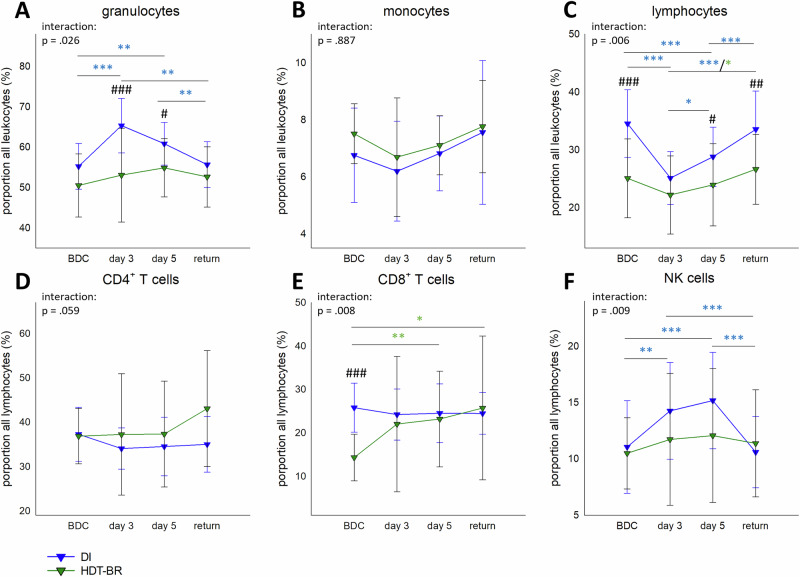
Changes in leukocyte proportions during DI and HDT-BR. Proportions of granulocytes (**A**), monocytes (**B**) and lymphocytes (**C**) among all leukocytes in whole blood as well as of CD4^+^ T cells (**D**), CD8^+^ T cells (**E**) and NK cells (**F**) among all lymphocytes at BDC, day 3, day 5 and return within 5 days DI (blue, n = 19) or HDT-BR (green, *n* = 12), respectively. Plots represent mean values ± SD over the courses of observation times. Data was analyzed with mixed ANOVA followed by Bonferroni post hoc test in the case of significant interaction. Differences between groups: ^#^*P* < 0.05, ^##^*P* < 0.01, ^###^*P* < 0.001. Differences between time points within the same group: *P < 0.05, **P < 0.01, ***P < 0.001, whereby differences in the DI group are indicated in blue and in the HDT-BR group in green asterisks.

Alterations in leukocyte subset proportions were detected for both the DI and the HDT-BR groups. In HDT-BR, only a slight and non-significant increase in granulocyte proportions was measured at day 5 (Fig. 4A), while proportions in the DI group showed a significant interaction with strongly higher values at day 3 and day 5 (Figs. 1A and 4A, Supplementary Table 2), that reached significant increase to the HDT-BR group (Fig. 4A). Of note, enhanced granulocyte levels in DI-participants at day 3 were accompanied by a mean 1.52-fold increase of IL-8 plasma concentrations which however declined again over study course (BDC: 1.52 ± 0.83 ng/ml; DI3: 1.96 ± 1.31 ng/ml; DI5: 1.1 ± 0.91; R + 1: 1.13 ± 0.62 ng/ml). Alterations in IL-8 plasma levels were not detected in HDT-BR^20^.

Monocyte proportions were similar in both groups (Fig. 4B). Lymphocyte proportions showed a significant interaction and were generally higher in the DI group. Proportions displayed a significant drop at day 3, which recovered to BDC values at return (Fig. 4C, Supplementary Table 2). In the HDT-BR group, such shifts occurred to a very weak extent only with a significant decline at day 3 compared to the return time point (Fig. 4C). Proportions of CD4^+^ T cells were unaffected by microgravity simulation (Fig. 4D). CD8^+^ T cell proportions were lower at BDC in the HDT-BR group than in the DI group, but rose then significantly within the study course (Fig. 4E). Proportions of NK-cells showed a significant interaction with pattern similar to granulocytes. During DI, NK cells numbers rose significantly until day 5 (Supplementary Table 2) while this effect was only weak in the HDT-BR group (Fig. 4F).

**Fig. 5:**
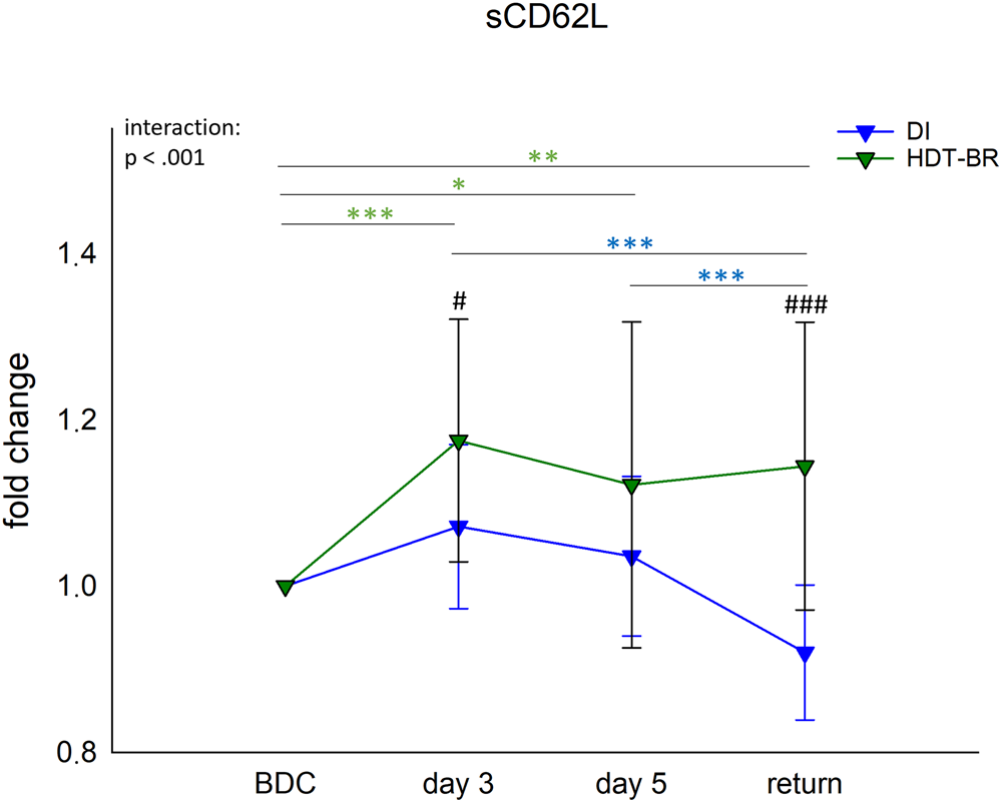
sCD62L concentrations in plasma. Changes in plasma concentrations of sCD62L within 5 days DI (blue, *n* = 19) and HDT-BR (green, *n* = 12) expressed as fold change to BDC. Plots represent mean values ± SD over the courses of observation times. Data was analyzed with mixed ANOVA followed by Bonferroni post hoc test. Differences between groups: ^#^*P* < 0.05, ^###^*P* < 0.001. Differences between time points within the same group: *P < 0.05, **P < 0.01, ***P < 0.001, whereby differences in the DI group are indicated in blue and in the HDT-BR group in green asterisks.

The comparison of sCD62L concentrations showed significant interactions in both groups, however with two different courses in DI and HDT-BR. While concentrations peaked at day 3 of DI and declined afterwards to values below BDC levels at return, they rose at day 3 of HDT-BR and remained at a high level until the end of observation period (Fig. 5, Supplementary Table 2).

### Stress hormones

The continuous increase in noradrenaline concentrations, as observed in DI (Fig. 3B) was not detectable in HDT-BR, where levels were constantly low (Table 8). Consequently, concentrations were significantly lower in HDT-BR than in DI at day 3 and day 5. Levels of urinary adrenaline concentrations were comparable in both groups and unaffected by microgravity simulation (Table 8).

**Table 8:** Urinary concentrations of the catecholamines noradrenaline and adrenaline in the course of 5 days DI (*n* = 19) and HDT-BR^12^

	DI				HDT-BR			
	BDC	day 3	day 5	return	BDC	day 3	day 5	return
Noradrenaline	34.59± 14.22	43.86± 16.23	44.64± 15.79	47.10± 19.22	31.33± 7.99	30.27± 13.89	28.47± 6.34	36.61± 9.02
Adrenaline	6.72± 4.31	7.16± 5.74	8.36± 4.65	7.22± 3.61	4.23± 1.64	5.55± 3.19	5.14± 2.87	5.58± 2.74

Values are given as mean ± SD.

### Fluid shift and hypovolemia

**Fig. 6:**
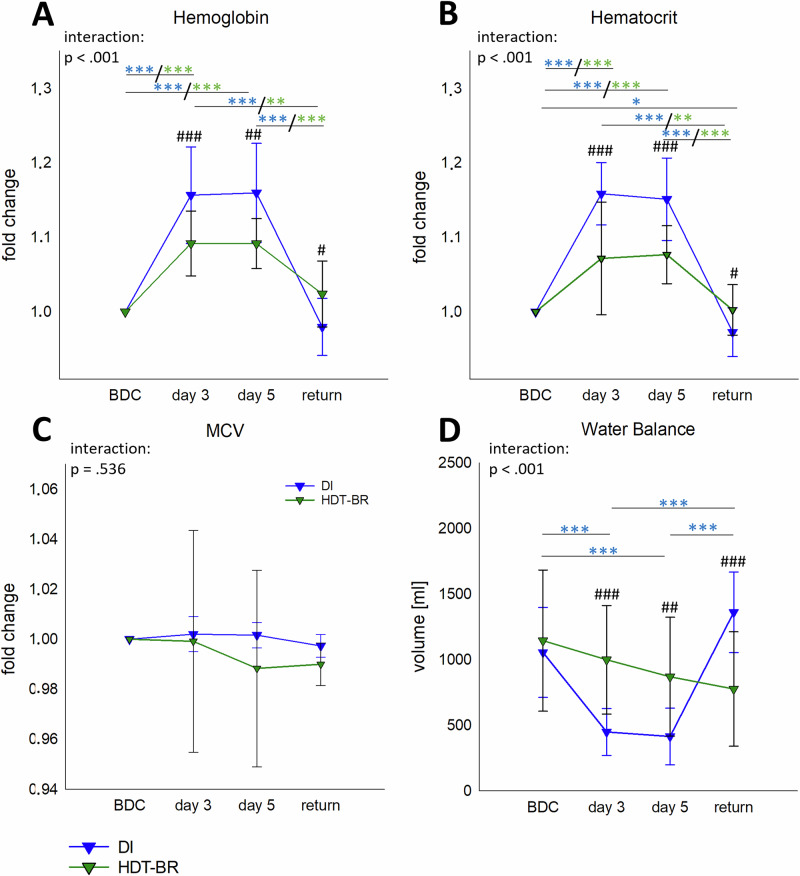
Fluid shift parameters. Blood parameters hemoglobin (**A**), hematocrit (**B**) and mean corpuscular volume (MCV; **C**) as well as water balance (**D**) at BDC, day 3, day 5 and return of 5 days DI (blue, *n* = 19) or HDT-BR (green, *n* = 13), respectively. **A**–**C** are expressed as fold change to BDC. Plots represent mean values ± SD over the courses of observation times. Data was analyzed with mixed ANOVA followed by Bonferroni post hoc test in case of significant interaction. Differences between groups: ^#^*P* < 0.05, ^##^*P* < 0.01, ^###^*P* < 0.001. Differences between time points within the same group: *P < 0.05, **P < 0.01, ***P < 0.001, whereby differences in the DI group are indicated in blue and in the HDT-BR group in green asterisks.

Hemoglobin and hematocrit values showed significant interactions in both groups and were increased at day 3 and day 5 of microgravity simulation. The increases were clearly stronger in DI than in HDT-BR (Supplementary Table 2), which resulted in significant differences between the two groups (Fig. 6A, B). Changes in PV were −24.8 ± 5.9 % (day 3) and −23.3 ± 7.4 % (day 5) during DI, and −12.1 ± 10.0 % (day 3) and −13.9 ± 4.4 % (day 5) in HDT-BR. MCV was unchanged in both groups (Fig. 6C). All values remained, despite these alterations, in physiological ranges.

A slight, but continuous decline in positive water balance was documented in HDT-BR, while there was a significant interaction in DI with a drop at day 3 and day 5, which then recovered quickly at return (Supplementary Table 2). Significant differences in water balance between the DI and HDT-BR groups occurred at day 3, day 5 and return. In HDT-BR water intake and urine output sank simultaneously at day 3 and day 5, leading to only moderate alterations in water balance^20^. During DI however, water intake was remarkably reduced while urine output volumes remained constant (Fig. 6D, Table 7).

## Discussion

The *Integrative Study of Physiological Changes Induced by a 5-Day Dry Immersion in Healthy Female Volunteers* (VivalDI I) was initiated to investigate the impact of simulated weightlessness by dry immersion (DI) on physiology and mental health in women^15^. In order to compare observed effects to those in men, VivalDI was expanded by the inclusion of male participants in follow-up investigations under the same set-up (VivalDI II). As part of the VivalDI study, the presented substudy was performed to obtain insights into immunological effects by five days DI. In addition, data obtained from investigations in male study participants allowed to identify differential impacts of five days DI and head-down tilt bed rest (HDT-BR) on inflammation state and fluid shifts by comparing to historical data sets of the BRAG-2 study^20,21^. The overall changes in immune state as observed during DI were independent of biological sex. The comparative analyses in males exposed to DI and HDT-BR indicate that fluids shifts were the primary drivers for the intervention-related differences reported herein.

Changes in the immune state induced by DI have been scarcely studied to date. Berendeeva and colleagues performed experiments on the immune state during a 7-day DI and observed increased leukocyte numbers but unchanged immune capacities after stimulation of isolated PBMCs at day 7 compared to pre and post DI^23^. Results derived from a 5-days DI study on the other side indicate reduced activation capacities of monocytes due to downregulation of various Toll-like receptors (TLRs) during DI^24^. However, low subject numbers and high variabilities between the individual study subjects did not allow for reliable conclusions. In a recent study, adaptive transcriptomic changes in T cells have been identified in the course of a 21-day DI^17^. In the present study, DI-related effects were compared in women and men. DI was shown to induce pronounced shifts of immune cell subset counts and proportions, as well as a subclinical immune cell activation early in observation period. Granulocytes and NK cell numbers showed both in women and in men a marked increase at day 3 and day 5 respectively, accompanied by a proportional decrease in T cell numbers. However, aside from known sexual dimorphisms in cell counts of monocytes, T-helper cells and NK-cells^12,25^, no sex-specific deviations were observed that could have been caused through DI.

To obtain first insights into functional immune responses during DI, viral loads of the latent viruses Epstein-Barr-Virus (EBV) and Torque Teno virus (TTV) as suitable surrogate marker correlating with the status of the hosts’ cell mediated immunity^26–28^, were quantified at BDC and R+1. While viral load of EBV was only occasionally changed in both groups, TTV shedding was increased in a higher percentage of female than male study participants at R+1. TTV is regarded as a human commensal virus with prevalence rates of up to 90 % and a high turn-over rate of virions^27,29^. Thus, changes in immune state can be monitored by increase in TTV load even over short time frames^27^.

Seeking for factors responsible for the observed immune state alterations during DI, we measured concentrations of psycho-neuroendocrine stress parameters as putative modulators of immune functions. Levels of the steroid hormones cortisol, DHEA and DHEA-S remained stable during observation period and values were highly comparable in female and male study participants. However, concentrations of the catecholamine noradrenaline displayed an increase within the study course and additionally, sex-specific patterns. Noradrenaline is a product of the central and peripheral adrenergic system^30,31^ and released in response to stress^32^. By binding to α- and β-adrenergic receptors expressed on the surfaces of leukocytes, noradrenaline is capable to immediately hamper immune cell functions^32^. The strong increase of noradrenaline concentrations at R+1 in females might thus represent one possible explanation for the higher viral loads in 66.6 % of female participants in the present study.

On the other side, release of noradrenaline is also known to increase cell numbers and proportions of both granulocytes and monocytes^33,34^ which would, however, be in discordance with high noradrenaline concentrations and simultaneous drop in cell numbers at R+1 in the present study. In addition, concentrations of all measured stress hormones remained in low physiological ranges, further disagreeing with immunological changes due to neuroendocrine stress responses in this study.

The increased levels of noradrenaline might be rather traced back to its stabilizing action on blood pressure by its’ vasoconstructive activity^30^. DI, same as HDT-BR, induces a redistribution of body fluids, reduced plasma volume, and subsequent changes in cardiovascular hemodynamics commonly leading to orthostatic intolerance^15,16,35^, a symptom that is frequently observed in astronauts when returning from microgravity conditions back to Earth^36,37^. In order to avoid hypotension in situations of altered body fluid volumes and orthostatic stress, noradrenaline levels increase^36^. Women generally show a higher susceptibility to orthostatic intolerance than men^13,15,38^ and it was already concluded from previous bed rest studies that women have different regulatory mechanisms for cardiovascular stability than men^35,38,39^. These observations correspond with the differentially rising noradrenaline courses in the present study, suggesting that males continuously readjust noradrenaline levels to maintain blood pressure in an adequate range, whereas females display a sudden increase at R+1 to assure orthostatic tolerance.

Adrenaline levels stayed constant over time in both groups with well-known higher concentrations in males than in females^31^. The lack of concentration increase of adrenaline over time might be explained by a potential exhaustion of its biochemical precursor noradrenaline to maintain blood pressure^36^.

Another explanatory approach for altered immune states still involves fluid shifts, but with an association to perceived hypovolemia during DI. Under induced hypovolemic conditions, such as lower body negative pressure^40^ and head-up tilt^41^ granulocyte and NK cell numbers have been shown to be increased in peripheral blood. Analysis of the activation states of monocytes, T cells and granulocytes during the present DI study revealed only marginal and occasionally significant changes, being not surprising since the study participants did not show any signs of inflammation. But still, changes in surface marker expression were detected as well as subclinical, however still enhanced plasma cytokine abundances, which can be debated in two directions. The first suggests the reduced plasma volume to result in an up-concentration of the present cytokines in blood plasma. Proportionally to plasma volume changes, cytokine abundance was continuously enhanced by 16 % in average in females and by 21 % in males, which might have also had an activating impact on surrounding immune cells. Plasma volume reductions of approximately 15 % under whole-body microgravity-simulating protocols were also reported by others^16,20^. The second explanatory approach likewise suggests fluid shifts, however with an association to hydrostatic pressure as a mechanical stimulus. In a cyclic hydrostatic pressure model, macrophages have been demonstrated to display pro-inflammatory properties^42,43^. This could also account for monocytes sensing positive pressure between the interstitial space and blood vessels in DI and subsequently affect other immune cells such as T cells. Altered rigidity of tissues and others substrates, which may occur in response to tissue swelling after hydration, was likewise shown to affect functions and pro-inflammatory activities in macrophages and T cells^42–44^.

However, if this apparent inflammation-resembling state can be attributed to up-concentration of blood plasma or activation by hydrostatic pressure cannot be finally evaluated at this point. A combination of both factors cannot be ruled out, especially considering that the increase in cytokine concentration in males, whose monocytes are more inflammatory in nature than in females^45^, appeared proportionally higher than plasma volume decrease. At this point one has to also resume on the slightly higher levels of some cytokines (although still very low) in females than in males measured here. Similar to immune cell counts and proportions, concentrations of circulating cytokines likewise display sex-specific differences by nature. For instance, baseline cytokine levels of the IL-1 family are higher in females than in males^46,47^. On the other side, the data situation for other cytokines is not as clear and dependent on the individuals’ condition^48,49^. If different baseline levels of cytokines may elicit sex-specific immune dysregulation in space or under spaceflight conditions differentially will be a matter of future investigations. Functional tests in Earth-bound spaceflight analogues demonstrated similar reactive immune capacities in women and men^14^.

Throughout the whole study water balance remained positive, however it strongly declined during DI compared to BDC or R+1 values, which was caused by reduced fluid consumption but constant urine output. During DI, fluids within the body are shifted to the upper body, leading to an increased venous return to the right heart which is physiologically recognized and transmitted as hypervolemia. As a consequence, a decrease in plasma volume is initiated that is finally expressed as reduced water intake^15,16^. While these effects were remarkably pronounced by DI, differences were not recognized between females and males.

Based on overlapping data collected within two studies using the whole-body microgravity protocols DI or HDT-BR for 5 days, the impact on leukocyte proportions and granulocyte activation, as well as on catecholamine levels and fluid shift was compared in two male study cohorts. This additional comparison was thought to serve as a preparatory proof-of concept investigation for future enrollment of female participants in such comparative studies. Current knowledge on the impact of HDT-BR on immunity derives from very heterogeneous studies of different durations, sampling time points, and in study populations. Kelsen and colleagues have for instance identified discrete alterations in cell mediated immunity that might have been induced within 21-day HDT-BR^50^. Findings obtained from 60-day HDT-BR show a maintained B cell homeostasis. Interestingly, in this study IL-10 and TNF levels have been shown elevated at day 3, which then declined again and stayed low until the end of the study^51^. One HDT-BR study even focused on immune effects in women and revealed a more efficient antibody production in response to immunization, when the HDT-BR intervention was combined with an exercise countermeasure^52^.

Both in DI and in HDT-BR leukocyte proportions were altered. However, a significant interaction occurred only in the DI group. Together with the high effect size for body fluid indicators, these observations are suggested to be again entailed by fluid shifts and intervention-associated differential levels of intravascular fluid loss. Moreover, the adhesion molecule CD62L was shed from granulocytes in both studies and detected in its soluble form in plasma which is commonly regarded as an indicator for inflammatory immune responses or immune cell-endothelium interactions^53^. Referring to results from the present investigations and from Feuerecker et al.^20^, no signs for inflammatory processes emerged in both studies. Consequently, and as already shown by in vitro studies^54,55^ as well as concluded by Feuerecker et al.^20^, a non-inflammatory and fluid-shift-related “mechanical” shedding of CD62L occurred. This conclusion is also supported by the observation that increased sCD62L levels corresponded inversely with decrease in water balance in both models. If the intensity of CD62L shedding is attributed to differences in fluid shift modalities or if higher levels in the HDT-BR group were caused by different sensitivities of the assay used, requires final clarification.

While no changes in adrenaline and dopamine levels were measured, noradrenaline concentrations rose continuously during DI but remained stable in HDT-BR. Plasma volume loss was more pronounced in DI than in HDT-BR and comparison of water balance analyses revealed an intense water loss in DI compared to HDT-BR.

The basis for these differences lies most probably in the main distinct property of these two models, which is support deprivation^56^. Since almost the whole body of the study subjects is surrounded by water, high levels of hydrostatic compression are induced during DI, leading to neutralization of internal pressures, a head-ward fluid shift^18^ and a uniform distribution of the subjects’ body weight leading to complete supportlessness^16,56^.

In HDT-BR on the other side, study subjects lay in bed in a 6° head-down tilt position, providing a head-ward fluid shift because of jugular vein congestion as well as higher venous and arterial blood pressures in the head and the neck^18^. Different than in DI, effects of gravity are not entirely removed but rather altered over the entire body from anterior to posterior, leading to compression of a large body surface area^18,57^.

Thereby caused putative differences in blood pressures or adaptations to maintain blood pressure in a physiological range may lead to different noradrenaline release patterns. Moreover, the intense differences in plasma volume loss in these two models reflect the differential dynamics and depth of changes within DI and HDT-BR as it has been already described by others^17,18,35,56^. It was shown, that effects occur up to seven times faster in DI than in HDT-BR^56,58^.

We acknowledge the limitations that because of operational and logistic challenges resulting in – though well in the margins of the manufacturers recommendations – different storage lengths of fixated whole blood within the two DI studies, analysis of surface marker was impeded, especially for monocytes in women. Besides emerging variations in n-numbers, a direct comparison was hence not performed sex-specifically and limited to DI-induced alterations. Lower n-numbers for study participants’ viral load analyses were due to insufficient saliva volumes for some study participants. Regarding the comparison of two DI and HDT-BR studies we acknowledge the limitations of different subject numbers and kits used for sCD62L quantification. However, possible batch-related discrepancies were overcome by data normalization to BDC values.

In conclusion, no sex-specific alterations in immunological states were observed within the present 5-days DI study. No conclusion can be drawn on the link to higher TTV shedding in females. For the examination of non-acute but chronic changes, DI studies of longer duration shall be considered. Lack of sex-specific effects in real spaceflight conditions has been already indicated by Drudi and Grenon^38^, however, both conclusions might yet be incomplete as by the lack of functional tests.

However, women and men seem to have differentially acting adaptation mechanisms to maintain blood pressure in times of hypovolemia induced by fluid shifts. In the range of the present investigations, alterations of immunological parameters and psycho-neuroendocrine mediators can be traced back to different modalities and intensities of fluid shifts that occur both in DI and in HDT-BR.

To evaluate differences in immune cell capacities between females and males during DI and HDT-BR regardless of fluid re-distribution, functional test shall be performed in future comparative studies of the same length.

## Supplementary information

Supplementary information

## Data Availability

Supplementary information accompanies this manuscript and is attached as a single file. The original data can be made available upon reasonable request.
