# Supplementary material for: Fluid shifts are main drivers for microgravity simulation-induced immune-physiological changes: findings from the VIVALDI studies
Source: NPJ Microgravity. 2026 Feb 16;12:15. doi: 10.1038/s41526-025-00555-z (PMC12914047; doi:10.1038/s41526-025-00555-z)
Supplement: Supplementary file 1 — Supplementary information [file 41526_2025_555_MOESM1_ESM.pdf]

Supplementary Material for

Fluid shifts are main drivers for microgravity simulation-induced immune-physiological changes: findings from the VIVALDI studies

Dominique Moser, Marie-Pierre Bareille, Angelique van Ombergen, Marion Hoerl, Federico D´Amico, Matthias Feuerecker, Christopher Dächert, Sandra Matzel, Adrien Robin, Nastassia Navasiolava, Marc-Antoine Custaud, Alexander Choukér on behalf of the Members of the VivalDI -study and Dry Immersion expert group

Correspondence to
Alexander Choukér: achouker@med.uni-muenchen.de

This file includes
Supplementary Figure 1
Supplementary Figure 2
Supplementary Figure 3
Supplementary Table 1
Supplementary Table 2

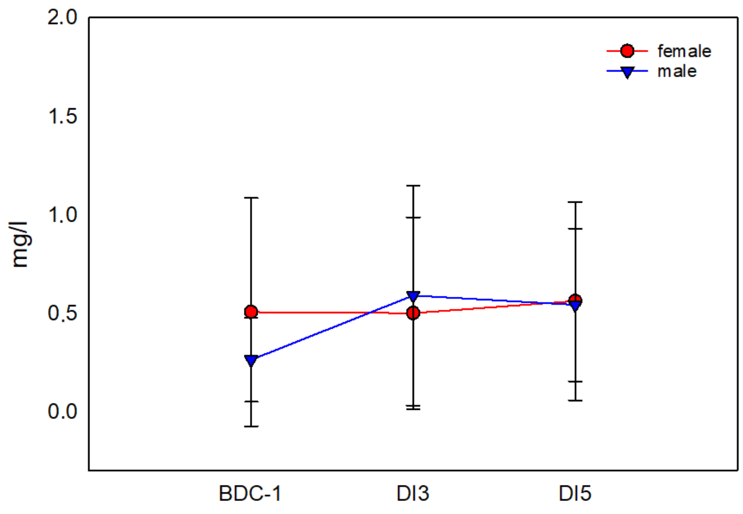

Supp. Figure 1 Serum CRP (C-reactive protein) concentrations (mg/l) at BDC-1, DI3 and DI5. Values are given as mean ± SD. Females: n = 18; males: n = 19.

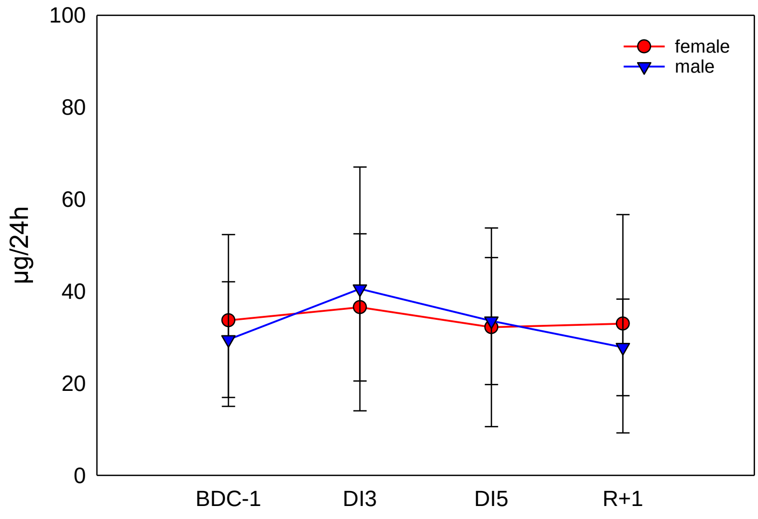

Supp. Figure 2 Urinary free cortisol (µg/24h) at BDC-1, DI3 and DI5 and R+1. Values are given as mean ± SD. Females: n = 18; males: n = 19.

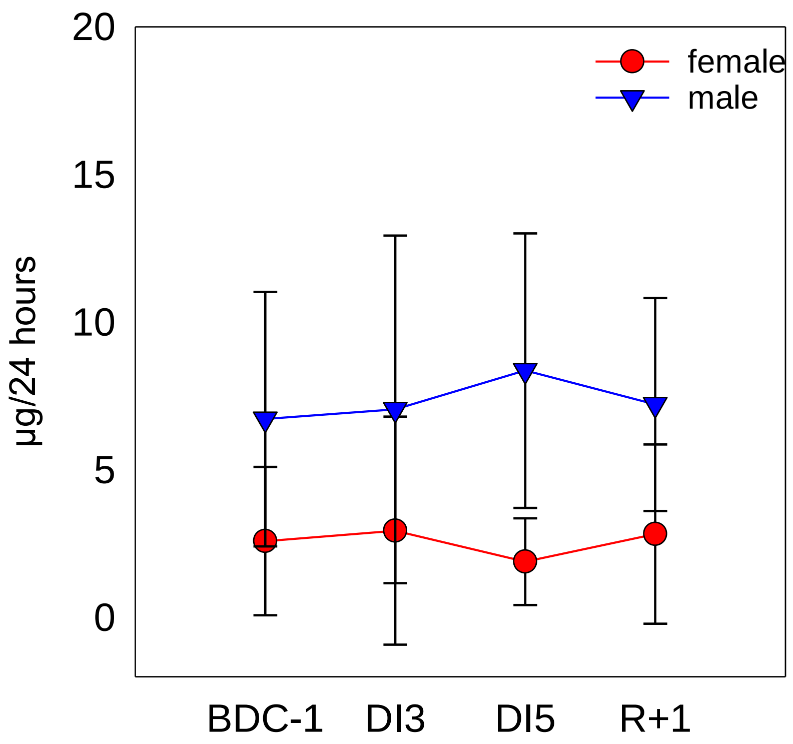

Supp. Figure 3 Urinary adrenaline concentrations. Plot represent mean values ± SD in females (red, n=18) and males (blue, n=19) over the courses of observation times. Single missing values were estimated by linear interpolation.

Supp. Table 1 Significant interaction, main effect terms as well as p-values for group and pre-post differences for investigations on sex-specific differences during dry immersion. Cohen´s d shows effect size for significantly different pairwise comparisons over time. Interaction is indicated in bold. Abbrev. F: female; m: male.

Interaction *F*(df1,df2)=F, *p-value*	η^2_p	Group difference (p-value)	Cohen´s d	Pre-Post differences	*P* - value	Cohen´s d
**Figure 1 - Granulocytes**						
F(2.69,94.07)=4.77 *p=.005*	0.120	*.958*	-	f: DI3 – R+1	*.006*	|0.52|
				m: BDC – DI3	< *.001*	|1.48|
				m: BDC – DI5	< *.001*	|1.21|
				m: DI3 – DI5	*.002*	|0.89|
				m: DI3 – R+1	< *.001*	|1.49|
				m: DI5 – R+1	< *.001*	|1.16|
**Figure 1- Lymphocytes**						
F(2.85,99.70)=5.03 *p=.003*	0.126	*.958*	-	f: BDC – DI3	*.023*	|0.75|
				m: BDC – DI3	< *.001*	|1.60|
				m: BDC – DI5	< *.001*	|1.22|
				m: DI3 – DI5	*.002*	|0.93|
				m: DI3 – R+1	< *.001*	|1.39|
				m: DI5 – R+1	< *.001*	|1.00|
**Figure 1- NK cells**						
F(3.00,105.00)=2.87 *p=.040*	0.076	BDC: *.005*	|1.00|	f: BDC – DI3	< *.001*	|1.21|
		DI5: *.001*	|1.16|	f: BDC – DI5	*.003*	|0.90|
		R+1: *.047*	|0.68|	f: DI3 – R+1	< *.001*	|1.24|
				m: BDC – DI3	*.004*	|0.89|
				m: BDC – DI5	< *.001*	|1.35|
				m: DI3 – R+1	< *.001*	|1.09|
				m: DI5 – R+1	< *.001*	|1.39|
**Table 3 – Neutrophilic granulocytes**						
F(2.76,96.65)=4.58 *p=.006*	0.116	R+1: *.036*	|0.72|	f: BDC – DI3	< *.001*	|1.19|
				f: BDC – DI5	< *.001*	|0.99|
				f: BDC – R+1	*.002*	|0.89|
				m: BDC – DI3	< *.001*	|1.67|
				m: BDC – DI5	*.018*	|1.02|
**Figure 3 - Noradrenaline**						
F(2.66,93.08)=2.90 *p=.045*	0.077	*.327*	-	f: BDC – R+1	< *.001*	|1.08|
				f: DI3 – R+1	*.009*	|0.93|
				f: DI5 – R+1	*.001*	|0.89|
				m: BDC – DI3	*.014*	|0.62|
				m: BDC – DI5	*.046*	|0.72|
				m: BDC – R+1	*.009*	|0.85|

**Table 4 – $CD4^{+}$/$CD28^{+}$ T cells**						
F(2.84,93.83)=3.70<br>*p=.016*	0.101	DI5: *.046*<br>R+1: *.004*	|0.70| |1.06|	f: BDC – R+1	*.008*	|1.28|
**Table 4 – $CD8^{+}$/$CD69^{+}$ T cells**						
F(2.23,71.22)=3.46<br>*p=.032*	0.098	DI5: *.010*	|0.94|	m: BDC – DI5	*.007*	|0.66|
**Table 4 – $CD8^{+}$/$CD28^{+}$ T cells**						
F(2.99,98.59)=3.041<br>*p=.034*	0.084	DI3: *.014*<br>R+1: *.026*	|0.87| |0.75|	f: BDC – DI3<br>f: BDC – DI5<br>f: BDC – R+1	*.001*<br>*.007*<br>*.047*	|1.06| |1.04| |0.64|
**Table 7 – Hemoglobin**						
F(2.98,104.22)=5.04<br>*p=.003*	0.126	BDC: < *.001*<br>DI3: < *.001*<br>DI5: < *.001*<br>R+1: < *.001*	|2.96| |3.29| |3.19| |2.67|	f: BDC – DI3<br>f: BDC – DI5<br>f: BDC – R+1<br>m: BDC – DI3<br>m: BDC – DI5	< *.001*<br>< *.001*<br>*.010*<br>< *.001*<br>< *.001*	|3.44| |2.30| |0.82| |3.68| |2.68|
**Table 7 - Hematokrit**						
F(2.84,99.37)=4.90<br>*p=.004*	0.123	BDC: < *.001*<br>DI3: < *.001*<br>DI5: < *.001*<br>R+1: < *.001*	|2.63| |2.75| |2.65| |2.16|	f: BDC – DI3<br>f: BDC – DI5<br>f: BDC – R+1<br>m: BDC – DI3<br>m: BDC – DI5<br>m: BDC – R+1	< *.001*<br>< *.001*<br>*.019*<br>< *.001*<br>*.003*<br>*.015*	|3.51| |2.19| |0.70| |3.85| |2.82| |0.80|
**Table 7 - Water intake**						
F(2.66,93.12)=6.41<br>*p<.001*	0.123	DI3: *.003*	|1.06|	f: BDC – DI3<br>f: BDC – DI5<br>m: BDC – DI5	< *.001*<br>< *.001*<br>< *.001*	|1.58| |1.05| |1.66|
**Table 7 - Urine output**						
F(2.76,96.41)=9.02<br>*p<.001*	0.205	DI3: *.002*	|1.10|	f: BDC – DI3	*.006*	|0.84|
**Table 7 - Water balance**						
F(2.96,103.66)=2.98<br>*p=.036*	0.078	BDC: *.042*<br>R+1: *.017*	|0.69| |0.82|	f: BDC – DI3<br>f: BDC – DI5<br>m: BDC – DI3<br>m: BDC – DI5<br>m: BDC – R+1	< *.001*<br>< *.001*<br>< *.001*<br>< *.001*<br>*.029*	|1.39| |1.71| |1.64| |1.62| |0.74|

Supp. Table 2 Significant interaction, main effect terms as well as p-values for group and pre-post differences for investigations on group-specific differences during dry immersion (DI) or head-down tilt bed rest (BR). Cohen´s d shows effect size for significantly different pairwise comparisons over time.

**Interaction** ***F*(df1,df2)=F, *p-value***	η^2_p	**Group difference (p-value)**	**Cohen´s d**	**Pre-Post differences**	***P* - value**	**Cohen´s d**
**Figure 4 - Granulocytes**						
F(1.95,56.62)=3.92 *p=.026*	0.119	D3: *<.001*<br>D5: *.013*	|1.37| |0.98|	DI: BDC – D3<br>DI: BDC – D5<br>DI: D3 – R<br>DI: D5 – R	< *.001*<br>*.003*<br>*.002*<br>*.001*	|1.48| |1.21| |1.49| |1.16|
**Figure 4 - Lymphocytes**						
F(3.00,87.00)=4.49 *p=.006*	0.134	BDC:*<.001*<br>D5: *.035*<br>R: *.007*	|1.51| |0.81| |1.08|	DI: BDC – D3<br>DI: BDC – D5<br>DI: D3 – D5<br>DI: D3 – R<br>DI: D5 – R<br>BR: D3 – R	< *.001*<br>< *.001*<br>*.017*<br>< *.001*<br>< *.001*<br>*.044*	|1.60| |1.22| |0.93| |1.39| |1.09| |1.13|
**Figure 4 - CD8$^+$ T cells**						
F(1.94,56.10)=5.31 *p=.008*	0.155	BDC:*<.001*	|2.09|	BR: BDC – D5<br>BR: BDC – R	*.009*<br>*.021*	|0.66| |0.57|
**Figure 4 - NK cells**						
F(3.00,87.00)=4.07 *p=.009*	0.123	*.352*	-	DI: BDC – D3<br>DI: BDC – D5<br>DI: D3 – R<br>DI: D5 – R	*.008*<br>< *.001*<br>< *.001*<br>< *.001*	|0.89| |1.35| |1.09| |1.39|
**Figure 5 - sCD62L**						
F(2.99,86.77)=8.63 *p<.001*	0.229	D3: *.026*<br>*R: <.001*	|0.86| |1.80|	DI: D3 – R<br>DI: D5 – R<br>BR: BDC – D3<br>BR: BDC – D5<br>BR: BDC – R	< *.001*<br>< *.001*<br>< *.001*<br>*.035*<br>*.002*	|1.37| |1.54| |1.19| |0.63| |0.83|
**Figure 6 - Hemoglobin**						
F(3.00,87.00)=18.45 *p<.001*	0.389	D3: *<.001*<br>D5: *.006*<br>R: *.011*	|1.61| |1.09| |1.01|	DI: BDC – D3<br>DI: BDC – D5<br>DI: D3 – R<br>DI: D5 – R<br>BR: BDC – D3<br>BR: BDC – D5<br>BR: D3 – R<br>BR: D5 – R	< *.001*<br>< *.001*<br>< *.001*<br>< *.001*<br>< *.001*<br>< *.001*<br>*.002*<br>< *.001*	|3.49| |2.52| |4.11| |3.75| |1.42| |2.82| |1.13| |1.45|

<table>
<tr><th colspan="7">Figure 6 - Hematocrit</th></tr>
<tr><td>F(2.74,79.50)=16.45</td><td>0.362</td><td>D3: <.001</td><td></td><td>DI: BDC – D3</td><td>< .001</td><td>|3.75|</td></tr>
<tr><td>p<.001</td><td></td><td>D5: <.001</td><td></td><td>DI: BDC – D5</td><td>< .001</td><td>|2.70|</td></tr>
<tr><td></td><td></td><td>R: .024</td><td></td><td>DI: BDC – R</td><td>0.10</td><td>|0.80|</td></tr>
<tr><td></td><td></td><td></td><td></td><td>DI: D3 – R</td><td>< .001</td><td>|4.29|</td></tr>
<tr><td></td><td></td><td></td><td></td><td>DI: D5 – R</td><td>< .001</td><td>|3.78|</td></tr>
<tr><td></td><td></td><td></td><td></td><td>BR: BDC – D3</td><td>< .001</td><td>|0.98|</td></tr>
<tr><td></td><td></td><td></td><td></td><td>BR: BDC – D5</td><td>< .001</td><td>|1.89|</td></tr>
<tr><td></td><td></td><td></td><td></td><td>BR: D3 – R</td><td>.002</td><td>|0.90|</td></tr>
<tr><td></td><td></td><td></td><td></td><td>BR: D5 – R</td><td>< .001</td><td>|1.39|</td></tr>
<tr><th colspan="7">Figure 6 - Water Balance</th></tr>
<tr><td>F(3.00,87.00)=17.40</td><td>0.375</td><td>D3: <.001</td><td>|1.80|</td><td>DI: BDC – D3</td><td>< .001</td><td>|1.80|</td></tr>
<tr><td>p<.001</td><td></td><td>D5: .001</td><td>|1.33|</td><td>DI: BDC – D5</td><td>< .001</td><td>|1.64|</td></tr>
<tr><td></td><td></td><td>R: <.001</td><td>|1.59|</td><td>DI: D3 – R</td><td>< .001</td><td>|2.24|</td></tr>
<tr><td></td><td></td><td></td><td></td><td>DI: D5 – R</td><td>< .001</td><td>|2.57|</td></tr>
</table>
